# Supplementary material for: Bioplastic biodegradability shapes microbial communities in a coastal brackish environment
Source: ISME J. 2026 Mar 13;20(1):wrag052. doi: 10.1093/ismejo/wrag052 (PMC13070388; doi:10.1093/ismejo/wrag052)
Supplement: Supplementary_materials_wrag052 [file supplementary_materials_wrag052.zip › Pessi-et-al-2025-Suppl-Figs.docx]

Bioplastic biodegrability shapes microbial communities in a coastal brackish environment

Igor S. Pessi^1,2,3,#^, Eeva Eronen-Rasimus^1,2,#^, Pinja Näkki^1^, David N. Thomas^4,3^, and Hermanni Kaartokallio^1^

^1^Marine and Freshwater Unit, Finnish Environment Institute (Syke), Helsinki, Finland

^2^Department of Microbiology, University of Helsinki, Helsinki, Finland

^3^Helsinki Institute of Sustainability Science (HELSUS), Helsinki, Finland

^4^Department of Environmental Sciences, University of Helsinki, Helsinki, Finland

^#^Both authors contributed equally to this work

Igor S. Pessi: <https://orcid.org/0000-0001-5926-7496>

Eeva Eronen-Rasimus: <https://orcid.org/0000-0002-1749-0793>

Pinja Näkki: <https://orcid.org/0000-0003-2499-4751>

David N. Thomas: <https://orcid.org/0000-0001-8832-5907>

Hermanni Kaartokallio: <https://orcid.org/0000-0002-3650-4628>

**Corresponding author:** Igor S. Pessi

Marine and Freshwater Unit

Finnish Environment Institute (Syke)

Agnes Sjöbergin katu 2

00790 Helsinki

Finland

[igor.pessi@gmail.com](mailto:igor.pessi@gmail.com)

# Supplementary figures S1–S8

## Figure S1

Biodegradation rates of different bioplastic materials incubated *in vitro* for four weeks with seawater from the Baltic Sea.

## Figure S2

Temporal dynamics of microbial community structure in the *in situ* mesocosm. Principal coordinates analysis of Bray-Curtis dissimilarities of Hellinger-transformed counts of unique 60-bp *rpsB* sequences extracted from the metagenomic reads. Samples with
< 325 *rpsB* reads were not included.

## Figure S3

Microbial community structure across the *in situ* and *in vitro* metagenomes and metatranscriptomes. Principal coordinates analysis of Bray-Curtis dissimilarities of Hellinger-transformed counts of unique 60-bp *rpsB* sequences extracted from the reads. Samples with < 325 *rpsB* reads were not included.

## Figure S4

Taxonomic profiles at the domain level. Relative abundance is shown as the 10% trimmed mean across six sampling times and 3 or 5 five biological replicates (*in vitro* and *in situ* experiments, respectively).

## Figure S5

Taxonomic profiles at the class level. Consensus taxonomic profile based on 59 marker gene sequences extracted from the metagenomic reads. Relative abundance is shown as the 10% trimmed mean across six sampling times and 3 or 5 five biological replicates (*in vitro* and *in situ* experiments, respectively).

## Figure S6

Upset plot of genus co-occurrence across the biodegraded, non-biodegraded, and seawater metagenomes from the *in situ* mesocosm. Only genera with ≥ 0.5% relative abundance in each material were included.

## Figure S7

Microbial community structure in the *in situ* metagenomes in the functional space. Principal coordinates analysis of Bray-Curtis distances dissimilarities of the prevalence of metabolic traits extracted from metagenome-assembled-genomes.

## Figure S8

Prevalence of metabolic traits in the *in situ* metagenomes, defined as the proportion of metagenome-assembled genomes (MAGs) containing the trait relative to all the MAGs detected in a given sample (≥ 25% horizontal coverage).
